# Integrative analysis of coral plasticity and adaptations reveals key proteins driving resilience to changes in ocean carbonate chemistry

**DOI:** 10.1007/s42995-025-00321-w

**Published:** 2025-11-05

**Authors:** Xiangcheng Yuan, Ellias Y. Feng, Jingtian Wang, Lei Jiang, Tao Yuan, Hui Huang, Weihua Zhou, Jack Chi-Ho Ip, Wei-Jun Cai, Senjie Lin

**Affiliations:** 1 Laboratory of Tropical Marine Bio-Resources and Ecology; Guangdong Provincial Key Laboratory of Applied Marine Biology, South China Sea Institute of Oceanology, Chinese Academy of Sciences, Guangzhou, 510301 China; 2 Key Laboratory of Tropical Marine Biotechnology of Hainan Province, Sanya Institute of Oceanology, SCSIO, Sanya National Marine Ecosystem Research Station, CAS-HKUST Sanya Joint Laboratory of Marine Science Research, Sanya, 572000 China; 3 College of Environmental Science and Engineering, Ocean University of China, Qingdao, 266100 China; 4 State Key Laboratory of Marine Environmental Science, College of Ocean and Earth Sciences, Xiamen University, Xiamen, 361102 China; 5 Science Unit, Lingnan University, Hong Kong SAR, China; 6 School of Marine Science and Policy, University of Delaware, Newark, DE 19716 USA; 7 Department of Marine Sciences, University of Connecticut, Groton, CT 06340 USA

**Keywords:** Alkalinity enrichment, Coral, Translation efficiency, Ocean acidification, Protein isoelectric point (pI)

## Abstract

Understanding how corals adapt to changes in seawater carbonate chemistry is crucial for developing effective coral conservation strategies. Research to date has mostly focused on short-term experiments, overlooking long-term evolutionary effects. Here, we investigated the link between short-term stress responses and long-term genetic adaptations in the coral species *Porites pukoensis* through experiments under varying CO_2_ and alkalinity conditions. Our results showed that alkalinity enrichment significantly increased coral calcification rates by 35%-45% compared to high CO_2_ treatment, highlighting the potential of alkalinity enrichment to mitigate acidification impacts. Corals modulated relative expression levels of basic and acidic proteins in response to changes in seawater carbonate chemistry in the stress experiments. Genomic data revealed that this mechanism has been evolutionarily fixed in various organisms adapting to seawater carbonate chemistry. Additionally, both experimental and genomic results showed that extracellular matrix proteins, like collagen with von Willebrand factor type A domain, were modified in response to distinct carbonate environments. Molecular dynamics simulations and in-vitro experiments demonstrated that the structural stability of these proteins contributes to coral resilience under acidified conditions. This study established an integrated framework combining stress experiments, multi-omics analyses, molecular simulations, and in-vitro validation to identify key proteins involved in coral adaptation to acidification.

**Supplementary Information:**

The online version contains supplementary material available at 10.1007/s42995-025-00321-w.

## Introduction

Corals have endured significant fluctuations in seawater CO_2_ levels over geological time scales. For example, at the end of the Cretaceous period, atmospheric CO_2_ levels were at least five times, and possibly up to ten times, higher than today's levels (Veron 2008; Pandolfi et al. 2011). However, anthropogenically caused rapid increases of atmospheric CO_2_ in recent decades have accelerated alterations in seawater carbonate chemistry (Caldeira and Wickett 2003), leading to pH decreases in the ocean termed ocean acidification (OA) (Orr et al. 2005). While there is growing concern about the potential effect of OA on coral communities (Albright 2011) and bio-calcification (Cohen et al. 2009; Kleypas et al. 2006; Langdon and Atkinson 2005), coral mortality due to OA has not been as much investigated as coral bleaching linked to ocean warming (OW) (Hughes et al. 2017). Apparently, corals are more resilient to OA than to OW, and some corals can live in acidic environments but with compromised growth or calcification (Shamberger et al. 2014), raising questions about the mechanisms of coral phenotypic plasticity and adaptation to associated carbonate chemistry changes.

Phenotypic plasticity may offer corals a short-term solution to varying carbonate chemistry. Over time, these beneficial phenotypic responses can become genetically fixed through natural selection, a concept known as the plasticity-first hypothesis (Levis and Pfennig 2016), which posits that phenotypic plasticity serves as a crucial precursor to evolutionary adaptation. Understanding this relationship between coral phenotypic plasticity and long-term genetic changes is essential for predicting how corals will adapt to future ocean changing carbonate system. In the future, corals might continue to encounter a changing carbonate system, through natural migration to deeper waters (Martinez et al. 2021) or through artificial interventions such as ocean alkalinization technologies aimed at enhancing coral calcification (Albright et al. 2016; Feng et al. 2015; Renforth and Henderson 2017). The ability of corals to demonstrate phenotypic plasticity in response to these shifts will be crucial for their long-term survival and adaptation.

Coral phenotypic plasticity enables them to adjust their physiological, transcriptomic, and proteomic responses to changes in carbonate chemistry, allowing them to counteract pH decreases and maintain a stable internal environment (Brown et al. 2022). Previous studies have investigated coral responses to changes in seawater carbonate chemistry at the organismal (McCulloch et al. 2012), cellular (Cai et al. 2016; Schoepf et al. 2017) and molecular level (Bhattacharya et al. 2016; Cai et al. 2016; Schoepf et al. 2017). Transcriptomic studies have revealed differentially expressed genes (DEGs) involved in ion transport, metabolic pathways, cellular stress responses, and adaptation (Comeau et al. 2019; Dixon et al. 2020; Moya et al. 2012; Schoepf et al. 2017). For instance, skeletal organic matrix genes were shown to respond to acidification, accompanied by the downregulation of carbonic anhydrases (Moya et al. 2012). In addition to transcriptomes, the responses at proteomic level have been studied and they tended to focus on coral skeletal organic matrix (Drake et al. 2013a; Ramos-Silva et al. 2013; Takeuchi et al. 2016), bleached and diseased corals (Ricaurte et al. 2016; Ricci et al. 2019; Wong et al. 2021), coral larvae (Sun et al. 2022) and evolution of coral calcification (Conci et al. 2020). Proteomics often turns out to be inconsistent with transcriptomic profiles due to the complicated gene regulation mechanisms, including post-transcriptional and translational modifications (Karlsen et al. 2021; Mayfield et al. 2016; Waldbauer et al. 2012). In this study, proteomics was chosen for its closer alignment with phenotype (Karlsen et al. 2021; Mayfield et al. 2016; Waldbauer et al. 2012), providing a more direct understanding of functional processes. Previous proteomic studies showed that protein isoelectric points (pI) tuning is one of the mechanisms to maintain stability and functionality under changing environmental pH, by adjusting protein pI. In bacteria, for instance, proteins resistant to low pH often exhibit higher pI values, enabling them to remain positively charged under acidic conditions (Fedyukina et al. 2014). However, it remains unclear whether corals employ similar proteomic pI tuning in response to ocean acidification, particularly in both short-term and long-term adaptive processes. While there has been considerable research on short-term responses and phenotypic plasticity, the relationship between phenotypic plasticity and long-term genetic changes leading to adaptation has been less explored. This gap in knowledge limits our understanding of how short-term plastic responses translate into permanent evolutionary changes, which is crucial for predicting the resilience of species in the face of rapidly changing environmental conditions.

**Fig. 1:**
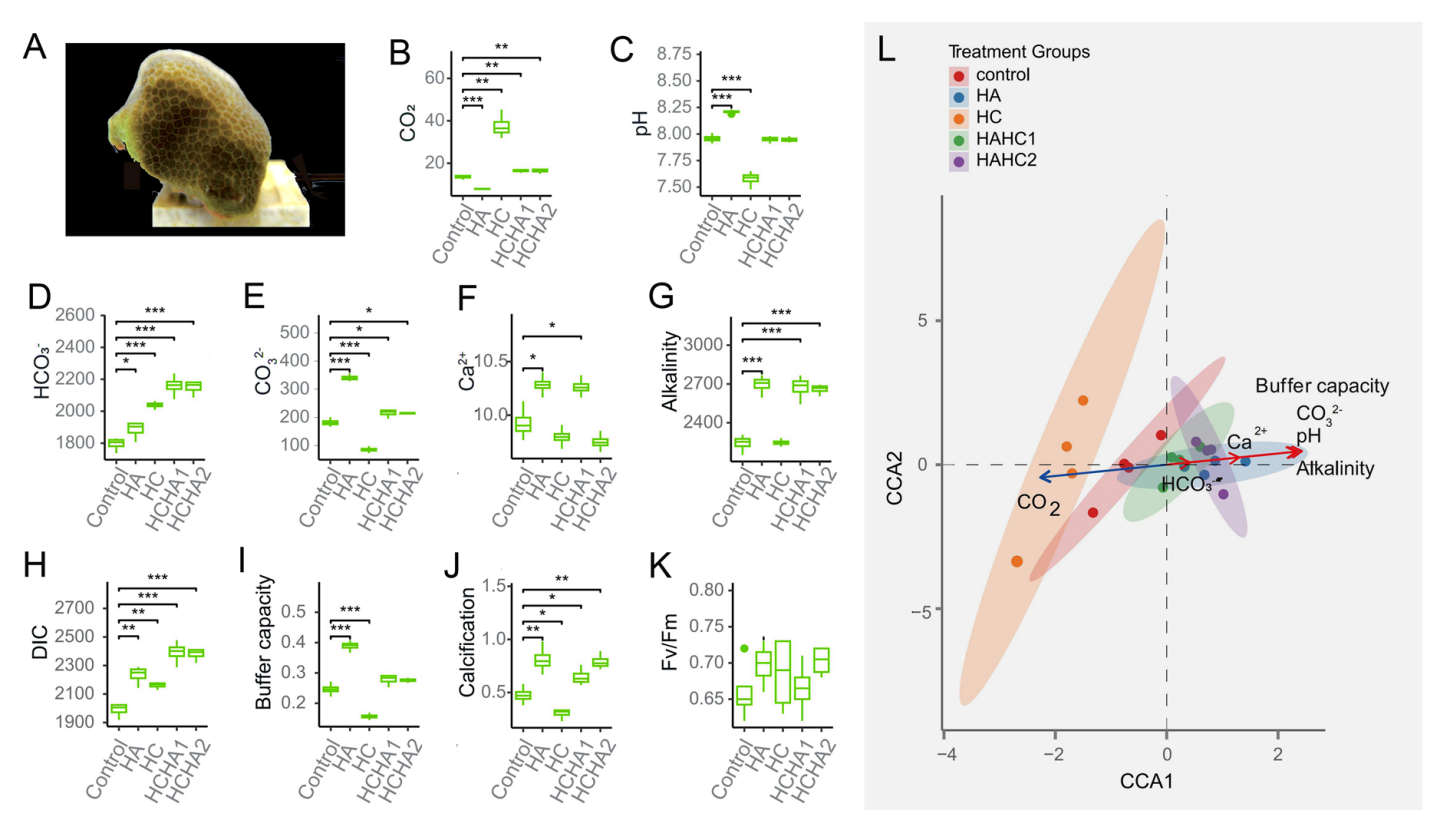
Experimental coral morphology and physiological responses to varying seawater carbonate chemistry. **A** Representative image of *Porites pukoensis* under one of the experimental treatments. The treatments include: control (natural seawater conditions), HA (high alkalinity with Ca(OH)_2_ solution enrichment), HC (high CO_2_ with CO_2_ enrichment), HCHA1 (high CO_2_ and alkalinity with CO_2_ plus NaOH enrichment), and HCHA2 (high CO_2_ and alkalinity with CO_2_ plus Ca(OH)_2_ enrichment). **B–K** levels of CO_2_ (μmol kg^−1^), pH, HCO_3_^−^ (μmol kg^−1^), CO_3_^2−^ (μmol kg^−1^), Ca^2+^ (mmol kg^−1^), alkalinity (μmol kg^−1^), dissolved inorganic carbon (DIC, μmol kg^−1^), buffer capacity of seawater, calcification rates (μmol cm^−2^ h^−1^) of coral at the end of experiment, Fv/Fm of coral at the end of experiment. **L** CCA analysis showing the relationship between calcification (response variable) and environmental drivers (CO_2_, pH, HCO_3_.^−^, buffer capacity). Arrows represent the gradients of environmental variables: longer arrows indicate stronger correlations with calcification, while their direction reflects the influence of each variable. Statistical significance denoted as: * (*p* < 0.05), ** (*p* < 0.01), *** (*p* < 0.001), *n* = 3

Here, we utilized physiological measurements, and proteomic profiling (Table S1), along with comparative genome analysis and molecular dynamic simulation analyses to study the coral responses to varying CO_2_ and alkalinity conditions. These conditions included normal CO_2_ (control), HC simulating high CO_2_ level expected in the year 2100 under the SSP5-8.5 scenario according to the IPCC (Canadell et al. 2021), HA simulating alkalinity enrichment into current seawater (Albright et al. 2016; Mongin et al. 2021), and HCHA treatments simulating alkalinity enrichment into future acidified seawater (Fig. 1A, Fig. S1). We focused on whether the phenomenon of proteomic pI tuning became fixed in the genomes as organisms adapted to their habitat pH conditions. Additionally, we aimed to investigate whether the observed proteomic variation pattern remains consistent across a diverse range of other organisms, and furthermore, to explore whether this pattern relates to coral resilience to acidification and coral biomineralization.

## Materials and methods

### Collection and cultivation

We selected the massive coral species *Porites pukoensis* (*P. pukoensis*) for our experiment because *Porites* is a prominent coral worldwide and *P. pukoensis* represents a dominant species in the Sanya reef area. Four colonies of the extensively distributed coral species *P. pukoensis* (with a diameter of 10–40 cm) from Hainan, China, were collected from Luhuitou Fringing Reef, Sanya Bay. The water was maintained at room temperature of 26 ± 0.1 °C and illuminated under a 12:12 h light–dark cycle (7:30 to 19:30) with ~ 300 ± 21 μmol photons m^−2^ s^−1^ irradiance (means ± SD) measured with a light sensor (LICOR-250, USA).

### Experimental design

The coral fragments (3–6 cm in diameter) were attached to a ceramic tile. In the experimental setup, ten tanks (0.5 m × 0.5 m × 0.5 m) were used, each containing eight coral fragments. After a 14 day acclimation period, corals were further acclimated for another 14 days in tanks with either CO_2_ enrichment or no enrichment (Fig. S1). To simulate high CO_2_ levels (~ 1349 μatm), a CO_2_ enricher (HC100B, Ruihua, China) was used to enrich the seawater. The tanks without CO_2_ enrichment were either left for no enrichment (i.e., control) or enriched with a Ca(OH)_2_ solution (i.e., HA). At the same time, the treatments of HCHA1 and HCHA2 were achieved through a 14-day CO_2_ enrichment, followed by the addition of NaOH or Ca(OH)_2_ for 35 days.

### Chemical measurements

We measured TA and pH values daily using a spectrophotometric procedure, which was calibrated against certified seawater reference material (Batch 118). Seawater CO_2_, HCO_3_^−^, CO_3_^2−^, and dissolved inorganic carbon (DIC) were calculated using the spreadsheet version of CO2SYS (Lewis and Wallace 1998). Ca^2+^ concentration was measured using a Ca^2+^ detection kit (Salifert Aquarium Products, Netherlands). Buffer capacity was estimated based on the method (Cai et al. 2016) as a measure of the seawater's ability to buffer pH. Samples for measuring alkalinity and pH at depths ranging from 0 to 1500 m were collected and analyzed with the same method mentioned above during a summer cruise in the South China Sea in 2012.

### Coral calcification rates and Fv/Fm

Coral buoyant weights for calcification estimation were measured at the beginning and end of the enriched cultivation period with an electronic balance (AUY220) (precision of ± 1 mg) (Spencer Davies 1989).

Maximum quantum yields of PSII (Fv/Fm) were measured in four replicates at the end of the experiment, using a Diving-Pulse Amplitude Modulated fluorometer (Walz GmbH, Germany). Fv/Fm is calculated as (Fm − F_0_)/Fm, where Fm represents the maximum fluorescence yield, and F_0_ denotes the initial fluorescence yield (Jiang et al. 2019).

### Proteomic analysis

At the end of the experiment, coral samples for proteome analysis were collected. Four replicates of coral fragments per treatment were immediately flash-frozen in liquid nitrogen for storage at − 80 °C until further processing. The protein extraction, digestion, and analysis were performed according to a previously described protocol (Sun et al. 2022). The raw MS data was analyzed using MaxQuant v1.5.3.17, employing label-free quantification. Quantifiable proteins were identified in at least two of the four biological replicates. Differential expression analysis of proteins (or DAPs) was conducted using the R package DEP (Zhang et al. 2018).

We calculated the pI of each predicted protein using the 'Peptides' package (Osorio et al. 2015). This approach enabled us to analyze the distribution of protein pI using protein sequences in the current study and two previous studies (Lin et al. 2022; Sun et al. 2022).

### Data analysis

Canonical Correspondence Analysis (CCA) was performed using the R package ‘vegan’ to evaluate the relationship between calcification and environmental variables using R package ‘ggplot2’. The package WGCNA (Langfelder and Horvath 2008) was used for undirected weighted co-expression correlation network analysis (WGCNA) to find key modules of the proteins. Gene Ontology enrichment analysis was performed using the R package ‘clusterprofiler’ (Yu et al. 2012) with a *p*-value cutoff < 0.05. Functional annotation was achieved by aligning the predicted protein sequences with eggNOG databases (Huerta-Cepas et al. 2019).

### Phylogenetic analysis, gene expansion, contraction, and Ka/Ks ratio

The protein sequences of bacteria, algae, sponge, cnidarians, snails and fish were downloaded from NCBI. OrthoFinder version 2.5.4 (Emms and Kelly 2019) was employed to identify homologous proteins. A species tree was generated through the Species Tree tool implemented using “iqtree” algorithm within OrthoFinder (Emms and Kelly 2019). The resulting tree was dated using r8s (Sanderson 2003). Gene expansion and contraction were analyzed using CAFE v.4.2 (De Bie et al. 2006) and OrthoVenn 3 (Sun et al. 2023) based on the species divergence time.

To investigate the molecular evolutionary process, synonymous (Ks) and non-synonymous (Ka) substitution rates were analyzed using ParaAT 2.0 (Zhang et al. 2012) and KaKs_calculator 2.0 (Wang et al. 2010). GO function enrichment was performed for the top 200 protein families exhibiting a high Ka/Ks ratio, using the clusterProfiler v3.10.1 (Yu et al. 2012). Functional annotation was achieved by aligning the predicted protein sequences with eggNOG databases (Huerta-Cepas et al. 2019).

### MD Simulations

Protein sequences were downloaded from NCBI, and protein structure prediction with AlphaFold 2.3.2 (Jumper et al. 2021). Protein pKa and structure at pH 6.5 and 8.5 was predicted using H +  + (Anandakrishnan et al. 2012). The MD simulations were performed using Gromacs with the AMBER force field, OPC water model, and 0.15 mol/L NaCl in a cubic water box. The full system was equilibrated for 2 ns with a time step of 1.0 fs.

### Expression of VWA and in-vitro CaCO_3_ precipitation

Because no suitable genomic resource for *Porites* was available, we used *Acropora digitifera* protein sequence (NCBI: XP_015767328.1) to design primers for *P. pukoensis*, which were artificially synthesized by Sangon Biotech (Shanghai, China). To obtain cell suspension from coral fragment, coral tissue was suspended in calcium-free artificial seawater with addition of a mixture of Dulbecco’s Modified Eagle Medium (Invitrogen) (Mass et al. 2012). The calcium carbonate precipitation experiments were carried out by adding suspended coral cells and 0.1 μmol l^−1^ of VWA to 5 ml artificial seawater (Instant Ocean Sea salt) for 14 days (Mass et al. 2012). The seawater pH was adjusted to pH 8.6 to mimic the pH environment found in coral calcifying centers with 0.05 M NaOH solutions (Sigma-Aldrich, USA). pH values were recorded using a Ross semi-micro-glass combination pH electrode (Orion) as a proxy for the relative amount of precipitate over a period of 2 days. The contents of the control and VWA added samples were subjected to centrifugation, and the resulting pellet was washed with Milli-Q deionized water and resuspended in 100% ethanol. Subsequently, crystal formation was examined using scanning electron microscope (S-3400N, Japan).

## Results

### Coral physiological responses to HC, HA and HCHA

HC treatment caused increases in CO_2_ levels, HCO_3_^−^ and dissolved inorganic carbon (DIC) concentrations and decreases in other parameters (*p* < 0.05), but showed no significant change in Ca^2+^, alkalinity and maximum photochemical efficiency (Fv/Fm) (*p* > 0.05) in seawater (Fig. 1B-K). HA treatment led to a decrease in CO_2_ levels but resulted in increases in other seawater carbonate chemical parameters compared to the control (*p* < 0.05) (Fig. 1B-I). The reversal of acidification effects on pH and CO_2_ was further observed in HCHA1 and HCHA2, in which a 14 day pre-incubation in HC was followed by 35 days of HA treatment using either Ca(OH)_2_ or NaOH enrichment (Figs. 1B-I).

HA and HCHA led to an increase in coral calcification rates, whereas HC resulted in a decrease (*p* < 0.05; Fig. 1J). However, changes in carbonate chemistry did not cause a significant change in Fv/Fm or bleaching (*p* > 0.05; Fig. 1K), indicating the resilience of the coral-zooxanthellae holobiont to the carbonate chemical changes, which is consistent with the findings in prior studies (Jiang et al. 2019). To gain insight into the relationship between carbonate chemical parameters and coral calcification rate, we conducted a Canonical Correspondence Analysis (CCA). Calcification rate negatively correlated with CO_2_ level, but positively correlated with CO_3_^2−^, buffer capacity, pH, alkalinity, and Ca^2+^ (*p* < 0.05; Fig. 1L).

### Proteomic responses to HA, HC and HCHA

**Fig. 2:**
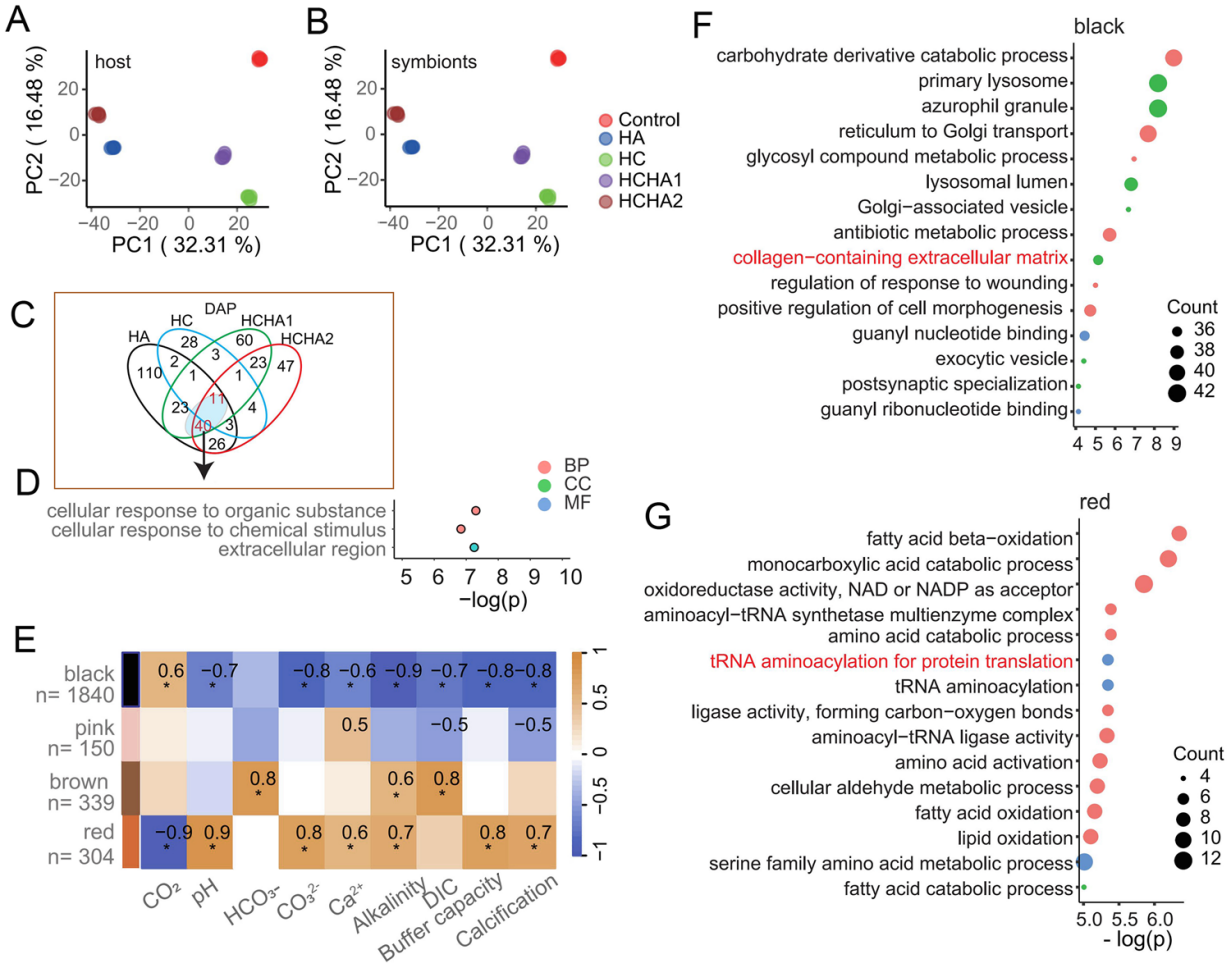
Coral and symbiont proteomes in responses to HC, HA and HCHA treatment. **A–B** PCA of host (**A**) and symbiont (**B**) proteins across treatments. **C** Overlap of host DAPs under different treatments. **D** Top GO terms for intersecting alkalinity-enriched samples. **E** WGCNA analysis with chemical parameters and calcification. **F–G** GO enrichment for black (**F**) and red (**G**) modules; “collagen ECM” and “tRNA aminoacylation” in red

Principal Component Analysis (PCA) was performed to visualize the variance in normalized data of protein abundance under various treatments (Control, HA, HC, HCHA1, and HCHA2). PCA plots for differentially abundant proteins (DAPs) in the coral host (Fig. 2A) and symbionts (Fig. 2B) demonstrated distinct clustering, indicating significant alterations in protein expression profiles in response to the treatments. Venn diagrams were constructed to examine the intersection of DAPs among the different treatment conditions. The intersections between DAPs within each treatment group (Fig. 2C) indicated a substantial overlap among these treatments. These results suggest that while some proteins are uniquely regulated by individual treatments, others are commonly affected by multiple conditions. Gene Ontology (GO) term enrichment analysis was conducted to elucidate the processes associated with the intersecting DAPs under alkalinity enrichment (HA, HCHA1, and HCHA2). The analysis of DAPs (Fig. 2D) revealed the top three enriched GO terms: "cellular response to organic substance" and "cellular response to chemical stimulus" and "extracellular region".

To probe deeper into the impacts of the experimental treatments on all 4,262 proteins in the coral host, Weighted Gene Co-Expression Network Analysis (WGCNA) was used to identify modules of co-expressed proteins and their correlations with various chemical parameters and calcification rates (Fig. 2E). The black module exhibited strong negative correlations with CO_2_ levels, while the red module showed strong positive correlations with alkalinity levels. These findings indicate distinct protein expression patterns associated with specific environmental conditions.

GO term enrichment analysis of proteins within the black module (Fig. 2F) revealed significant enrichment in processes such as "carbohydrate derivative catabolic process", "primary lysosome", and "collagen-containing extracellular matrix". Consistent with the results of DAPs analysis, the enrichment of this term also highlights the critical role of the extracellular region in the coral's response to environmental changes. In contrast, proteins within the red module (Fig. 2G) were enriched in terms related to "fatty acid beta-oxidation", "amino acid catabolic process", and "tRNA aminoacylation for protein translation," underscoring the metabolic adjustments in response to elevated alkalinity. These metabolic processes may potentially counteract the effects of elevated alkalinity by producing H^+^ ions (Fig. S2). Increased alkalinity can also stimulate activities in amino acids and translation processes, such as amino acid catabolic processes and tRNA aminoacylation for protein translation (Fig. 2G).

### Plasticity of corals to CO_2_ changes through modulating proteomic acidity in the coral host

**Fig. 3:**
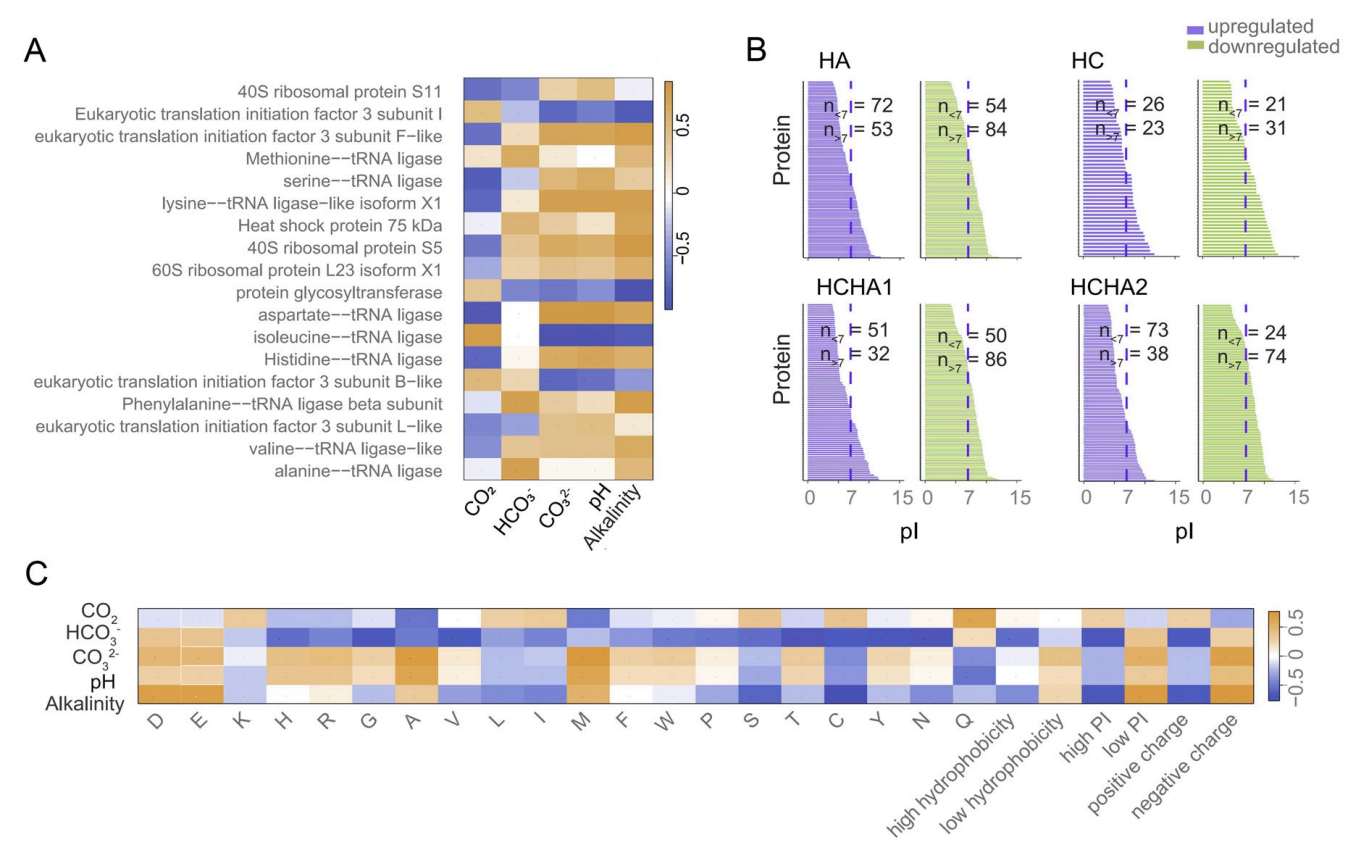
Effects of HC, HA and HCHA on protein pI in the coral. **A** Correlation between seawater chemistry (CO_2_, HCO_3_^−^, CO_3_^2−^, pH and alkalinity) and the abundance of proteins involved in translation. **B** Protein pI distribution in upregulated (red bars) and downregulated (green bars) DAPs in response to HA, HC**,** HCHA1**,** and HCHA2. n_<7_ and n_>7_ are the numbers of proteins with pI < 7 and > 7, respectively. **C** Relationship between seawater chemistry (CO_2_, HCO_3_^−^, CO_3_.^2−^, pH and alkalinity) and the abundance of proteins with a higher percentage of specific amino acids (top 1000), which are shown on the X-axis. D and E: proteins rich in acidic amino acids (aspartic acid and glutamic acid); K, H, and R: proteins rich in basic amino acids (lysine, histidine, and arginine); G, A, V, L, I, M, F, W, and P: proteins rich in nonpolar amino acids (glycine, alanine, valine, leucine, isoleucine, methionine, phenylalanine, tryptophan, and proline); S, T, C, Y, N, and Q: proteins rich in polar amino acids (serine, threonine, cysteine, tyrosine, asparagine, and glutamine)

As WGCNA analysis showed that translation was related to seawater chemistry, we conducted a correlation analysis to investigate the impact of CO_2_, HCO_3_^−^, CO_3_^2−^, pH and alkalinity on proteins that play roles in translation (Fig. 3A). Significant correlations were observed between the expression of some translation machineries and carbonate chemistry parameters (Fig. 3A). Particularly, the expression of eight out of the 20 amino acid-tRNA ligases exhibited a positive correlation with alkalinity (Fig. 3A). Upregulating specific tRNA ligases can selectively enhance the biosynthesis of proteins with certain amino acid compositions, especially those with charged amino acids (Crean et al. 2013; Mukai et al. 2008). This led us to postulate that the variations in proteins might result in a shift in protein homeostasis such as the acid/base-associated property pI. To investigate this hypothesis, we examined proteomic pI changes in response to the carbonate chemical treatments in the coral host. Results revealed that more alkaline proteins (characterized by a pI > 7) were downregulated in response to HC, HA, and HCHA treatments (Fig. 3B). Among upregulated proteins under HC, HA, and HCHA, a greater proportion of proteins were acidic than alkaline (Fig. 3B). In addition, there was a positive correlation between alkalinity and the abundance of proteins rich in acidic amino acids such as aspartic acid (D) and glutamic acid (E) (Fig. 3C). Consequently, higher alkalinity levels coincided with more increases in acidic proteins (pI < 7) and fewer basic proteins (pI > 7) (Fig. 3B). These results suggest that *P. pukoensis* regulated the relative synthesis levels of acidic proteins to modulate proteomic pI in response to acid/basic environmental conditions to curb intracellular pH swings.

**Fig. 4:**
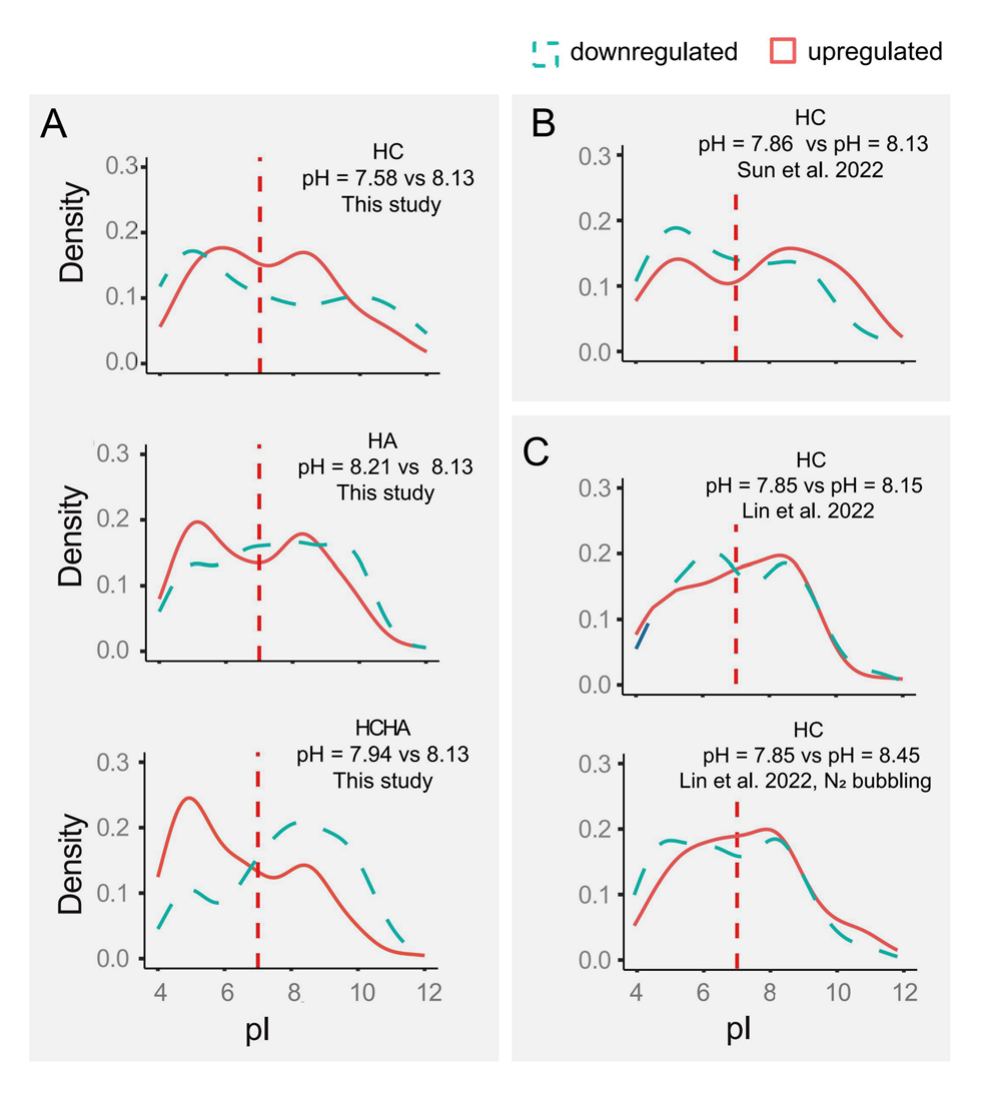
Effects of pH on coral protein pI distribution. **A** Comparisons of the pI values of DEPs across different pH levels: pH 7.58 vs. 8.13 (left), pH 8.21 vs. 8.13 (middle), and pH 7.94 vs. 8.13 (right). **B** Comparison of pI distribution between pH 7.86 and 8.13, based on data reported in Sun et al. (2022). **C** Comparisons between pH 7.85 and 8.15 (middle), as well as pH 7.85 and 8.45 (right), based on data from Lin et al. (2022)

To investigate whether coral responses to changes in carbonate chemistry universally involve the modulation of proteomic acidity, we conducted a comparative analysis of the current data along with two prior proteomic datasets that investigated the response of *Pocillopora damicornis* (PD) and *Galaxea fascicularis* to elevated CO_2_ (Lin et al. 2022; Sun et al. 2022). In contrast to the HC treatment (Fig. 4A), the HA and HCHA treatments led to a shift in the upregulated protein pI toward the acidic end, distinguishing them from the downregulated proteins (Fig. 4A). Similar to the findings in this study, two previous datasets showed that exposure to high CO_2_ resulted in a shift in upregulated protein pI toward the alkaline end, contrasting with the downregulated proteins (Fig. 4B, C).

### Persistence of short-term proteomic changes in long-term adaptation

**Fig. 5:**
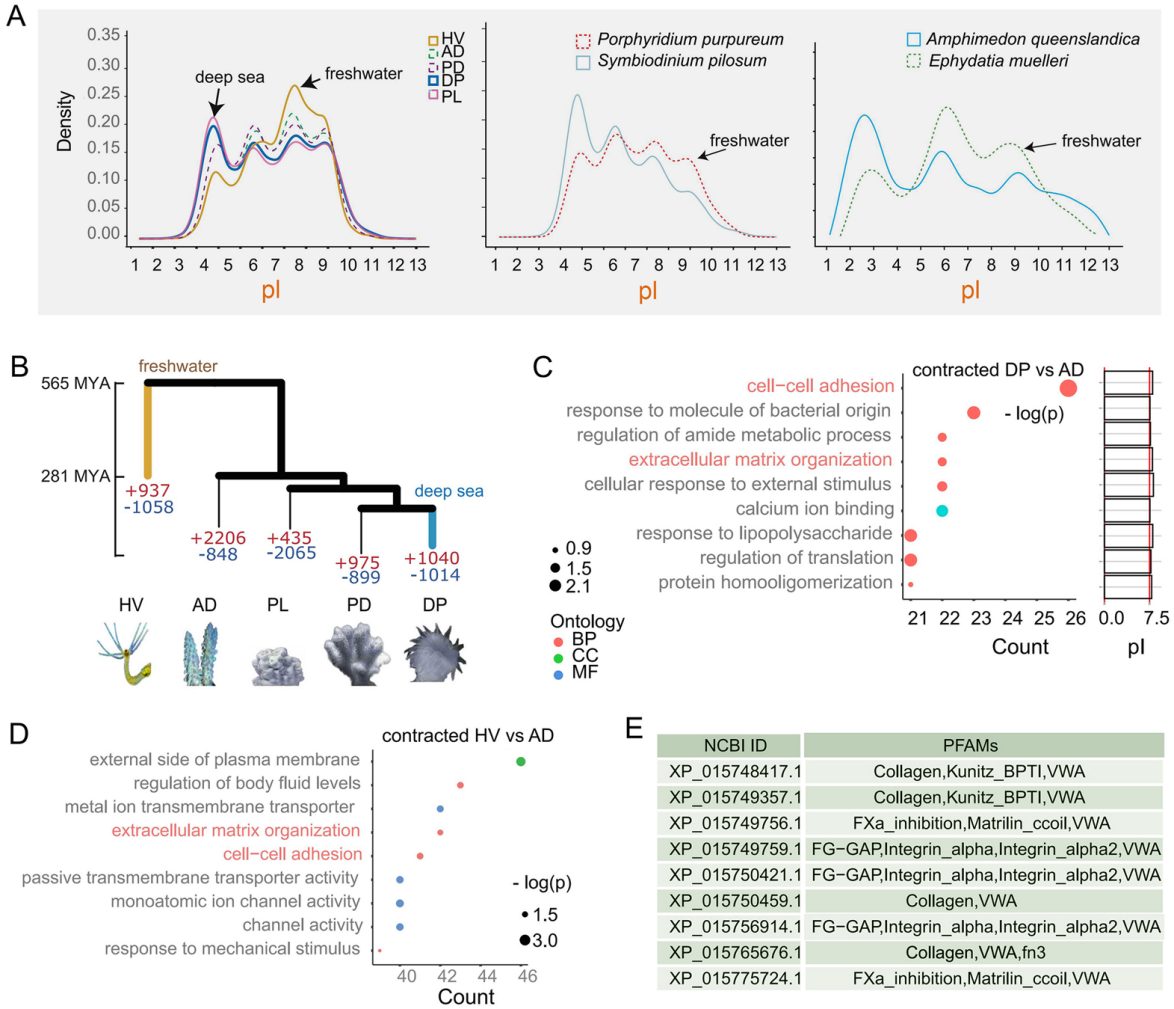
Cnidarian proteome shifts along carbonate chemistry gradients. **A** pI density distributions among cnidarians, algae, and sponges. **B** Phylogeny of expanded/contracted protein families. **C–D** GO terms for contracted families (DP vs AD with mean pI values; HV vs AD); ECM-related terms in red. **E** A list of contracted extracellular proteins containing VWA domain

The proteomic pI profile shifts observed in response to short-term seawater carbonate chemical changes led us to postulate that prolonged exposure of a marine organism to its acidic or alkaline habitat condition may result in the adaptive selection of certain protein pI profile in the genome. To address this hypothesis, we analyzed pI distributions, ke protein families, and evolutionary trends in corals and *Hydra* from different habitats (Fig. 5A). Compared to shallow waters, deep seawater typically has higher alkalinity and CO_2_ levels (Fig. S3). The varying depths result in unique carbonate environments suited for specific coral species. For instance, *Desmophyllum pertusum* (DP) inhabits in deeper waters (Herrera and Cordes 2023) characterized by a stable HCHA condition, compared to *Acropora digitifera* (AD), *Porites lobata* (PL), and PD that live in shallower waters akin to the control condition. In contrast, *Hydra vulgaris* (HV) lives in freshwater environments (pH ≤ 7) and represents an example of low pH and low alkalinity adaptation. Similar to some other freshwater species, HV exhibits a more abundant alkaline genome-encoded proteome with high pI (Fig. 5A), which may be associated with specific adaptations to freshwater environments. This mirrors the coral stress response to HC (Fig. 5A), which favors upregulation of alkaline proteins.

To further explore whether the potential adaptation to pH changes through the modulation of proteomic pI occurs in other organisms than cnidarians, we conducted an analysis of pI distributions in several taxonomic groups. These groups included bacteria, algae, sponges, snails, and fish (Fig. 5A, Fig. S3). A consistent pattern as the case of cnidarians was observed across the taxonomic groups of bacteria, microalgae, and sponges where species from freshwater environments exhibited an increase in alkaline proteins and a decrease in acidic proteins (Fig. 5A). The acidity of proteins was modulated by changes in the composition of charged amino acids within proteins (Fig. S4). For example, the elevated percentage of histidine and lysine in freshwater algae *Cyanidioschyzon merolae* resulted in a greater abundance of positively charged amino acids compared to other seawater red algae and coral symbionts (Fig. S4). In contrast, freshwater fish and snails did not upregulate alkaline proteins compared to their marine counterparts (Fig. S3), indicating that variations in carbonate chemistry have less pronounced effects on these evolutionarily advanced organisms.

In addition to protein pI evolution, we conducted an analysis of the genomic evolutionary among corals and Hydra (Fig. 5B-E). Consistent with our stress experiment showing the downregulated expression of extracellular proteins by HCHA (Fig. 2D, F), we observed a contraction in genes encoding extracellular proteins in DP and HV when compared to AD (Fig. 5B-D). These extracellular proteins exhibit an alkaline nature, with mean pI values > 7.5 (Fig. 5C), and often contain a von Willebrand factor type A domain (Fig. 5E). Moreover, the proteins with the highest Ka/Ks ratio are also extracellular proteins when the genome was compared between DP and AD, especially the proteins with VWA domain.

### Extracellular proteins were associated with resilience against acidification and biomineralization

**Fig. 6:**
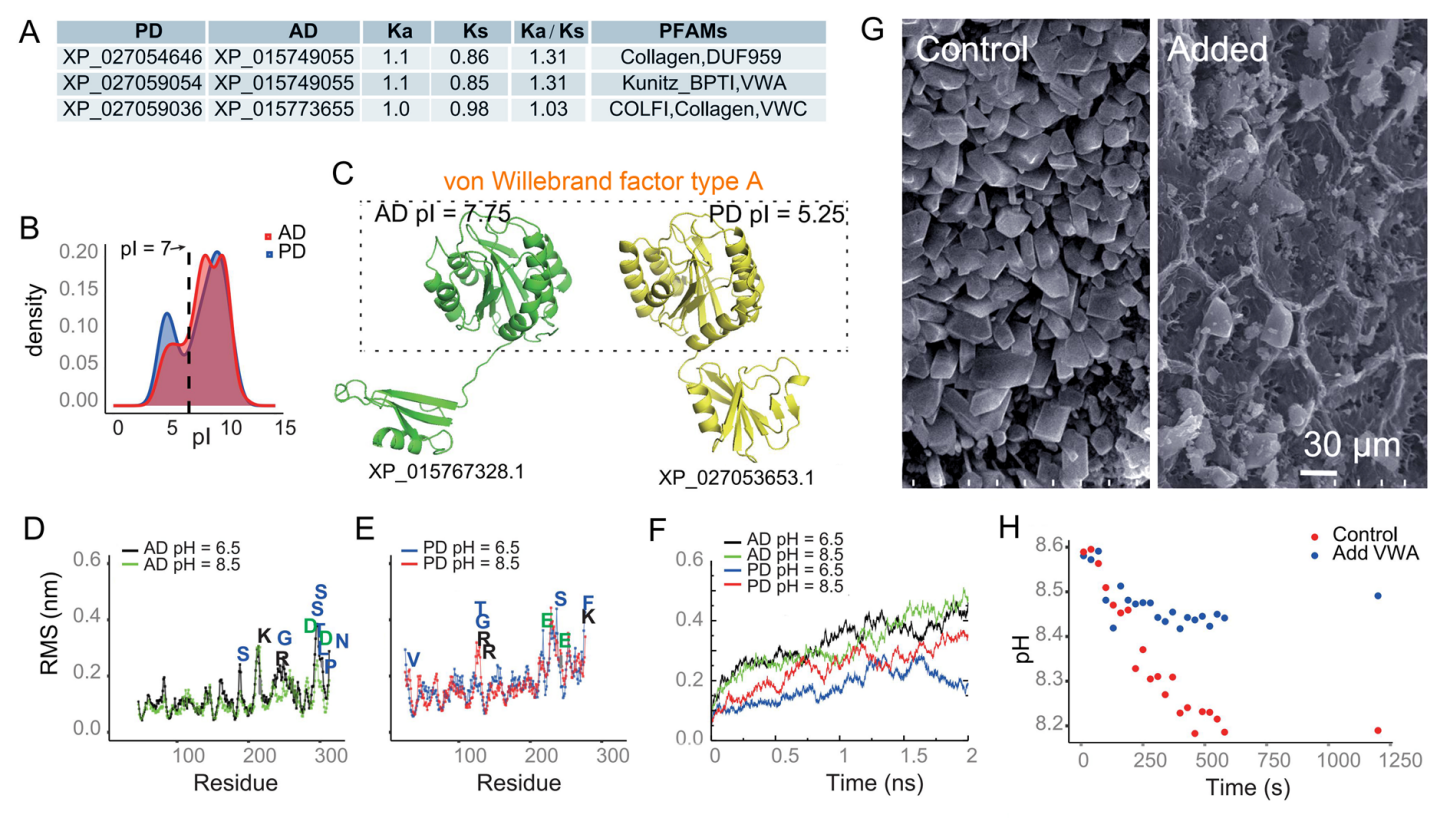
Collagen analysis, molecular dynamic simulation and in-vitro CaCO_3_ precipitation experiments in *A. digitifera* (AD) and *P. damicornis* (PD). **A** Table showing the protein IDs, Ka/Ks ratios, and associated Pfam (protein family) domains for collagen proteins in AD and PD. **B** Density plot showing the distribution of pI values for collagen proteins in AD (red) and PD (blue). **C** Structural comparison of collagen proteins from AD (XP_015767328.1) and PD (XP_027053653.1). The structures of the von Willebrand factor type domain are shown with respective pI values. **D, E** Root-mean-square fluctuation (RMSF) analysis of collagen alpha-6(VI) at pH 6.5 and 8.5. Residues with fluctuation differences greater than 0.1 nm are labeled for AD (**D**) and PD (**E**), respectively. **F** Root-mean-square deviation (RMSD) analysis of collagen alpha-6(VI) at pH 6.5 and 8.5 in both AD and PD. **G** SEM images of calcium carbonate precipitates obtained without (control) and with the addition of VWA domain. **H**, pH values over time during the CaCO_3_ precipitation experiments, comparing control and VWA added conditions

The genomics and short-term responses of variations in extracellular proteins in response to carbonate chemistry led us to hypothesize that these proteins may play a crucial role in coral resilience against acidification. To investigate this hypothesis, we analyzed gene Ka/Ks ratios (Fig. 6A), as well as gene loss, gain, expansion and contraction between PD and AD (Data Fig. S5). The comparison between PD and AD is because PD has been found to exhibit higher resilience against acidification compared to corals *Acropora* (Schoepf et al. 2013; Comeau et al. 2014, 2017). PD resilience has been attributed to its ability to maintain constant carbonate chemistry conditions in its calcifying fluid (Comeau et al. 2017; DeCarlo et al. 2018). Our analysis showed that genes encoding extracellular proteins were altered in PD through modulating gene Ka/Ks ratios and gene gains (Fig. 6A and Fig. S5). Among these extracellular proteins mentioned above, we found that collagen and protein with VWA domain were highly modulated (Fig. 6A), and there was more acidic collagen in PD (Fig. 6B).

Since our stress experimental results showed that extracellular proteins were differentially expressed (Fig. 2), we conducted molecular dynamic simulation to examine the stability of this collagen under different pH conditions in AD and compared it with the corresponding orthologous proteins in PD (XP_027053653.1) (Fig. 6C-F). RMSF profile revealed that the residues with high fluctuations contained charged amino acids such as K, R, D, and E (Fig. 6D, E). Protein RMSD values in PD were lower at both pH = 6.5 and 8.5 than the value in AD, indicating that overall protein structure in PD was more stable than in AD (Fig. 6F). These results suggest that collagen alpha-6 with VWA domain in PD is structurally stable in acidified seawater and this potentially contributes to PD's resilience against acidified seawater.

To study the role of VWA in biomineralization, we expressed VWA (the domain of XP_015767328.1) in *Escherichia coli* and conducted calcium carbonate precipitation experiments. The precipitates revealed that crystals formed with the addition of VWA exhibited a different shape compared to the control without additives (Fig. 6G). In addition, we measured the relative amount of CaCO_3_ precipitation by monitoring pH value decreases over time (Heinemann et al. 2011). CaCO_3_ precipitation experiment showed that the pH values decreased much less with the addition of VWA (Fig. 6H), indicating that VWA inhibited calcium carbonate precipitation. This finding aligns with the results of our stress experiments, where an alteration of extracellular proteins such as VWA in response to higher alkalinity led to higher calcification rates (Fig. 6H and 1J).

## Discussion

### Impacts of alkalinity enrichment on coral calcification

By manipulating seawater carbonate chemistry, our results demonstrated that alkalinity enrichment can mitigate the adverse impacts of OA on coral calcification rates. This finding aligned with previous research conducted at the coral community in a pseudo-atoll in the southern Great Barrier Reef (Albright et al. 2016) and coral *P. porites* studied in laboratory experiments (Marubini and Thake 1999), showing alkalinity enrichment increased coral calcification. However, we acknowledge that the small sample size (*n* = 4 genets), which is a common challenge in coral studies due to logistical and ethical constraints, limits the generalizability of our findings. Future studies with larger sample sizes and diverse genetic backgrounds are essential to validate these findings across different coral species and populations.

### Shifts in coral protein acidity-alkalinity properties

Furthermore, our experiment showed a shift in coral protein acidity-alkalinity properties: HA and HCHA increased acidic proteins with low pI values (Fig. 4). These findings align with previous research linking proteomic acidity variations to salinity in bacteria (Lanyi 1974; Elevi Bardavid and Oren 2012; Fedyukina et al. 2014). Halophilic bacteria synthesize more acidic proteins to overcome hindrance of protein hydration by high salt concentration (Elevi Bardavid and Oren 2012; Fedyukina et al. 2014). Alkalinity levels are typically closely correlated with salinity, which helps explain why the effects of HA and HCHA are similar to those of high salt concentrations. While previous studies have reported bacterial responses of protein acidity to changes in the acidity of surrounding water (Elevi Bardavid and Oren 2012; Fedyukina et al. 2014), our study represents the first report of modulation of protein acidity in corals and some other eukaryotes in response to changes in seawater carbonate chemistry.

### Role of extracellular proteins in pH homeostasis

Among these proteins that contribute to pI shift, extracellular proteins likely play an important role in regulating pH homeostasis. This is evidenced by the downregulation of many extracellular proteins (Fig. 2F), which were usually characterized by alkaline properties (Fig. 5C). In addition, we observed a decrease in genes encoding extracellular proteins in DP and HV compared to that in AD (Fig. 5B-D), with the proteins exhibiting the highest Ka/Ks ratio, particularly those containing VWA domain (Fig. 5E). DP, HV and AD represent organisms inhabiting in different conditions of carbonate chemistry. Their regulation of genes encoding extracellular proteins through gene family contraction and gene mutation further confirm that the modulation of these proteins is related to changes in carbonate chemistry (Fig. 5). This modulation potentially enables corals to buffer and maintain pH homeostasis in response to changes in seawater carbonate chemistry through regulating extracellular proteins.

### Extracellular proteins and coral calcification

In addition to maintaining pH homeostasis, certain extracellular proteins have been implicated in coral calcification. For instance, VWA domain has been identified as one of the most frequently utilized protein domains in biomineralization processes in invertebrate animals (Liu and Zhang 2021), and hypothesized to play a role in coral mineralization process as part of the skeletal organic matrix (Drake et al. 2013b). In addition to VWA domain, coral acid-rich proteins (CARPs) have been reported to bind Ca^2+^ and accelerate coral biomineralization (Mass et al. 2013). However, the specific roles of VWA in coral calcification have not been validated in previous studies. Our in-vitro precipitation experiments suggested that VWA domain may decrease coral biomineralization in coral cells (Fig. 6G, H). This findings is consistent with previous results indicating that coral skeletal organic matrix plays diverse roles in biomineralization (Reggi et al. 2016). It remains unclear how the VWA domain affects coral calcification but it could either indirectly regulate coral calcification through signaling pathways or act directly as a part of the mineralization matrix.

## Concluding remark

We reveal that corals fine-tune the abundance of proteins related to the translation machinery, such as aminoacyl-tRNA synthetases, to adjust protein pI values, particularly within the extracellular matrix, as a mechanism to maintain pH homeostasis. This mechanism seems to be widely present across a wide range of organisms inhabiting different aquatic environments, suggesting that the distribution of proteomic pI values in the genome reflects adaptation to specific habitat pH conditions. Furthermore, by analyzing the consistency between stress-induced proteomic responses and genomic changes, we highlight the role of extracellular proteins in promoting coral resilience to acidification. This observation supports the plasticity-first hypothesis (Levis and Pfennig 2016), which suggests that the phenotypic plasticity of proteomic pI adjustments may serve as a short-term strategy to preserve homeostasis. Over time, such plastic responses could lead to genetic accommodation, eventually resulting in the fixation of advantageous traits. These findings have certain implications for understanding the evolutionary mechanisms by which corals and other marine organisms adapt to rapidly changing oceanic conditions, particularly under the threats of OA. Identifying the key molecular players involved in pH regulation and adaptation can guide the development of more targeted conservation strategies.

## Supplementary Information

Below is the link to the electronic supplementary material.

Supplementary file1 (DOCX 2115 KB)

## Data Availability

Supplementary material for this article is available at Github (https://github.com/frandxc/HC-AE-effects-on-coral). Proteomic data are available via ProteomeXchange with identifier PXD048099.

## Declarations

### Research involving human and/or animals participants

We confirm that all relevant international, national, and institutional regulations for the sampling and experimental use of organisms in this study have been adhered to, and all required permissions have been secured.
