# Supplementary material for: Integrative analysis of coral plasticity and adaptations reveals key proteins driving resilience to changes in ocean carbonate chemistry
Source: Mar Life Sci Technol. 2025 Nov 5;7(4):1008–20. doi: 10.1007/s42995-025-00321-w (PMC12662967; doi:10.1007/s42995-025-00321-w)
Supplement: Supplementary file 1 — Supplementary file1 (DOCX 2115 KB) [file 42995_2025_321_MOESM1_ESM.docx]

**Supplementary Information**

**Integrative analysis of coral plasticity and adaptations** **reveals key proteins driving resilience to changes in ocean carbonate chemistry**

Xiangcheng Yuan^a,b^, Ellias Y. Feng^c^**^*^**, Jingtian Wang^d^, Lei Jiang^a,b^, Tao Yuan^a,b^**^*^**, Hui Huang^a,b^, Weihua Zhou^a,b^, Jack Chi-Ho Ip^e^, Wei-Jun Cai^f^, Senjie Lin^g^**^*^**

^a^ Laboratory of Tropical Marine Bio-resources and Ecology; Guangdong Provincial Key Laboratory of Applied Marine Biology, South China Sea Institute of Oceanology, Chinese Academy of Sciences, Guangzhou 510301, China

^b^ Sanya National Marine Ecosystem Research Station; CAS-HKUST Sanya Joint Laboratory of Marine Science Research; Key Laboratory of Tropical Marine Biotechnology of Hainan Province, Sanya Institute of Oceanology, SCSIO, Sanya 572000, China

^c^ College of Environmental Science and Engineering, Ocean University of China, Qingdao, 266100, China

^d^ State Key Laboratory of Marine Environmental Science, College of Ocean and Earth Sciences, Xiamen University, Xiamen, Fujian 361102, China

^e^ Science Unit, Lingnan University, Hong Kong SAR, China

^f^ School of Marine Science and Policy, University of Delaware, Newark, DE 19716, USA

^g^ Department of Marine Sciences, University of Connecticut, Groton, CT 06340, USA.

***Corresponding author:**

E-mail address:

Senjie Lin: senjie.lin@uconn.edu

Ellias Y Feng: [fengyuming@ouc.edu.cn](mailto:fengyuming@ouc.edu.cn)


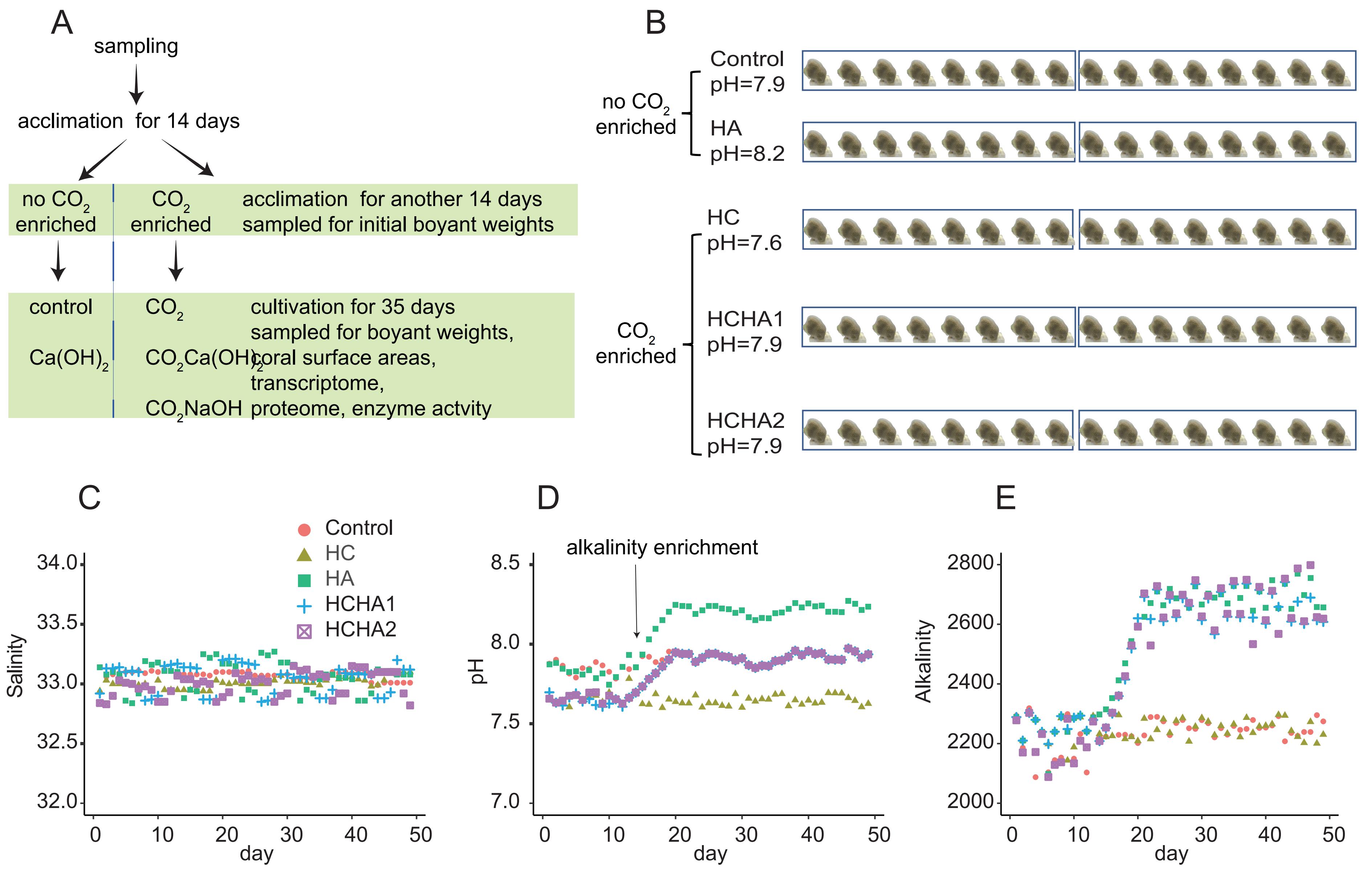


**Fig. S1**. **Experimental workflow, design and time series data. A** Acclimation and sampling timeline. After a 14-day acclimation period, corals were further acclimated for another 14 days in tanks with either CO_2_ enrichment or no enrichment. The initial buoyant weights of corals were measured. The tanks without CO_2_ enrichment were either left for no enrichment (i.e. control) or enriched with a Ca(OH)_2_ solution (i.e. HA) for 35 days. At the same time, the tanks with CO_2_ enrichment were either left without any further treatment (i.e. HC) or added with a Ca(OH)_2_ (i.e. HCHA1) or a NaOH solution (i.e. HCHA2) for 35 days. At the end of cultivation, samples were collected for analysis of buoyant weights, coral surface areas, and proteome, respectively. **B** Experimental design with pH values and treatments: control, HA, HC, HCHA1 and HCHA2 (n = 5 treatments × 2 tanks × 8 samples). Time series of seawater **(C)** salinity(‰), **(D)** pH and **(E)** alkalinity (μmol kg^-1^).


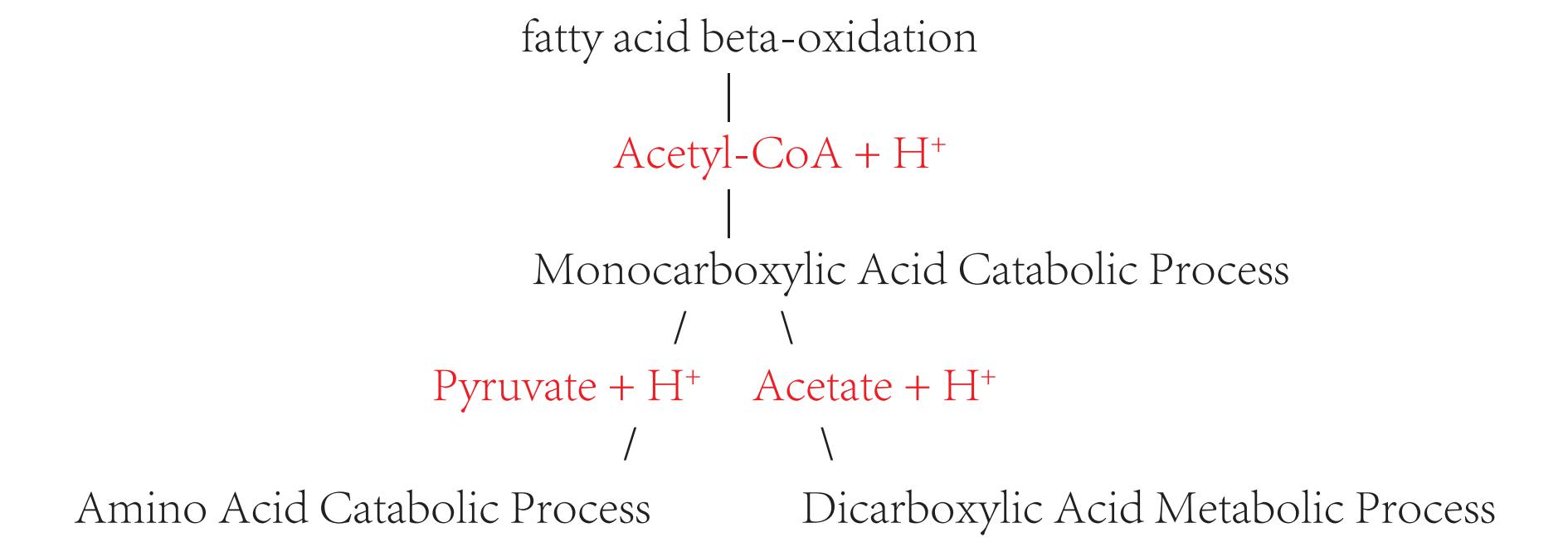


**Fig. S2**. **The possible connections between metabolic processes.** This enrichment indicates that elevated alkalinity levels upregulate these metabolic processes, some of which are marked in red to signify their production of H^+^ ions.
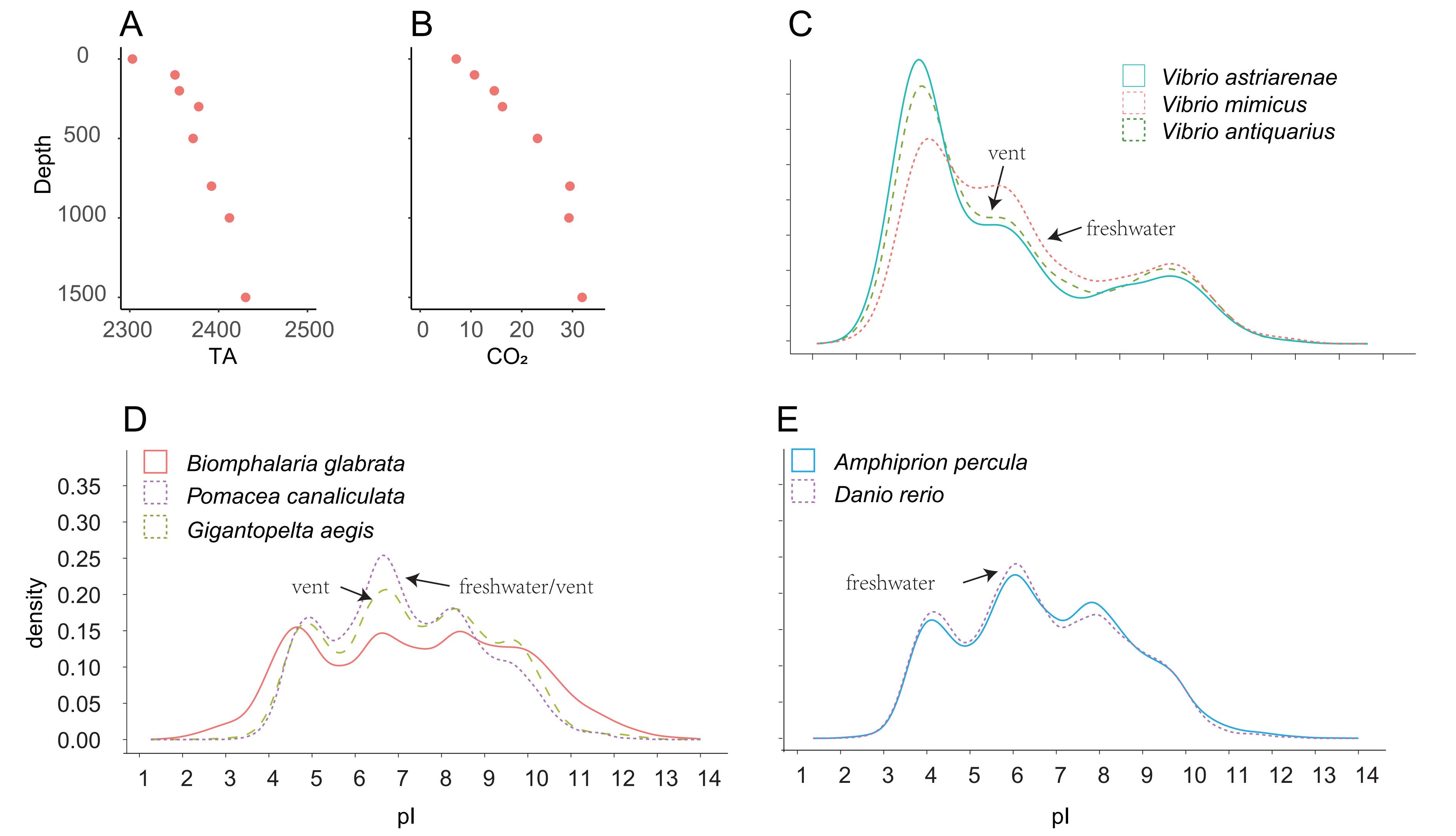


**Fig. S3**. **Genome-wide pI distribution of different marine organisms. A, B** Vertical distribution of alkalinity and CO_2_ levels in the South China Sea near Hainan Island where corals were collected. **C–E** pI density distribution in bacteria (*Vibrio astriarenae,* *Vibrio antiquaries,* and *Vibrio mimicus*), snails (*Biomphalaria glabrata, Pomacea canaliculata, Gigantopelta aegis*) and fish (*Amphiprion percula* and *Danio rerio*)*.* Solid lines connect organisms from seawater environments, while dashed lines link organisms from freshwater and deep-sea habitats.


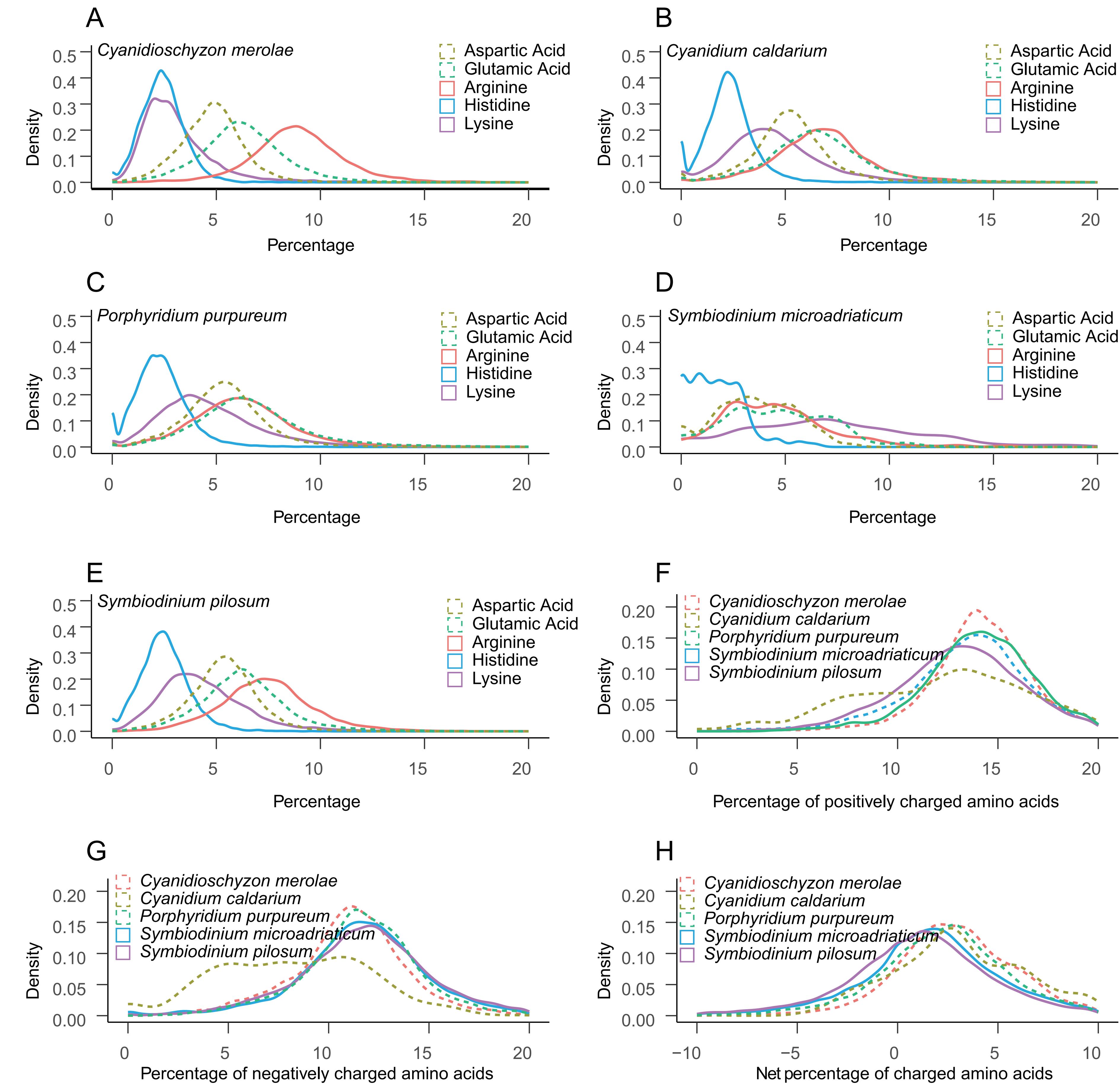


**Fig. S4**. **Charged amino acid composition in proteins of red algae and coral symbiont species in the genus *Symbiodinium*.** Density of percentage of aspartic acid, glutamic acid, arginine, histidine, and lysine in freshwater algae: **A–E** *Cyanidioschyzon merolae*, *Cyanidium caldarium* and *Porphyridium purpureum*; and in seawater algae: *Symbiodinium microadriaticum* and *Symbiodinium pilosum*. Solid lines represent acidic amino acids, while dashed lines represent basic amino acids. **F–H** Sum percentage of positively charged amino acids, negatively charged amino acids, and net percentage of charged amino acids (percentage of positively charged amino acids minus percentage of negatively charged amino acids). Solid lines represent organisms from seawater, while dashed lines represent organisms from freshwater.

**
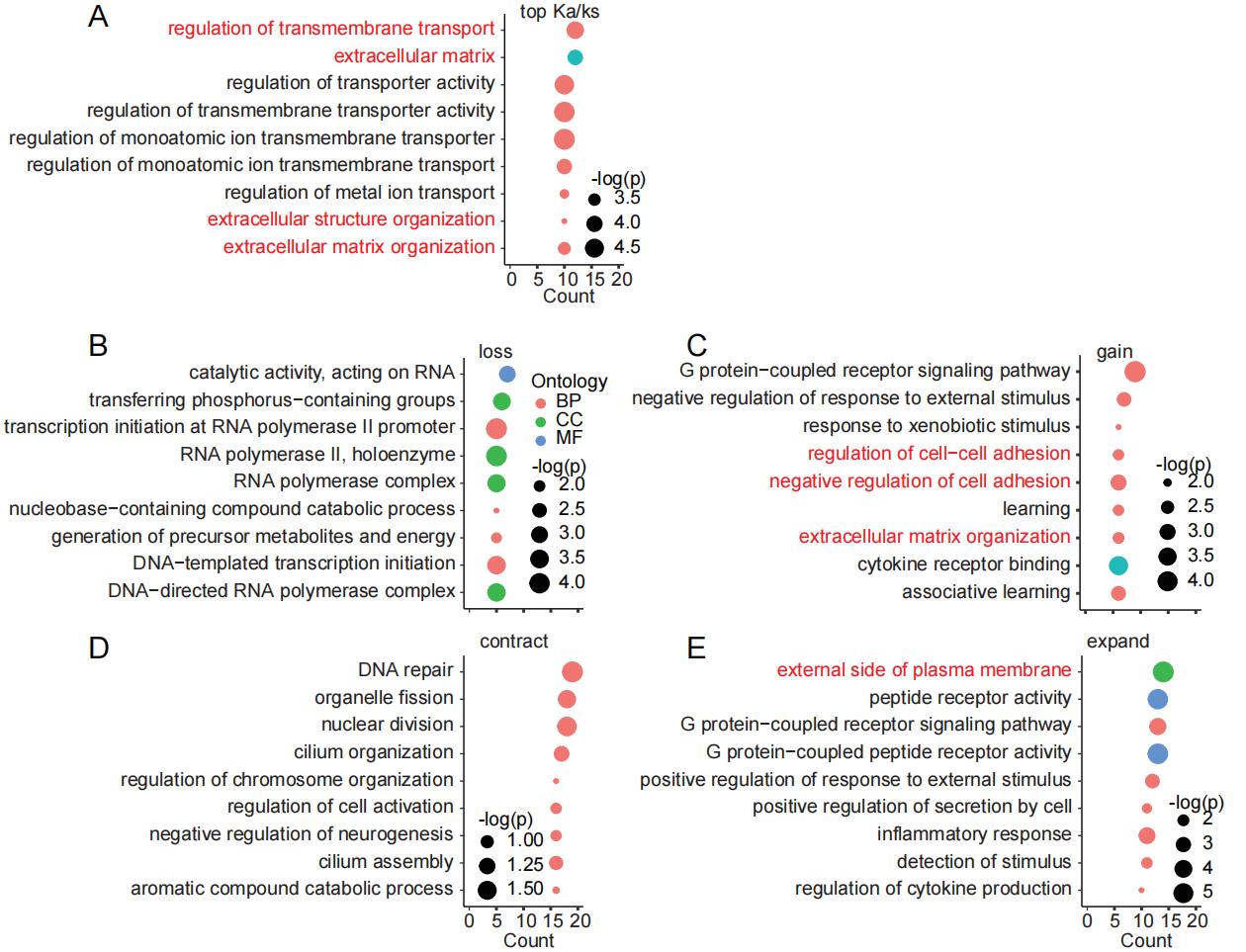
Fig. S5**. **Evolutionary shift in genome-derived protein profile between *P. damicornis* (PD) and *A. digitifera* (AD).** **A G**O terms of the top 200 proteins with highest Ka/Ks. Bubble size corresponds to the -log(p). **B–E** GO terms of the gene gain, loss, contracted and expanded protein families. BP: biological processes; CC: cellular components; MF: molecular functions. The terms related to extracellular matrix was highlighted in red.

**Table S1** Peptide and protein numbers from proteome sequencing.

| Sample | Peptide Num | Protein Num |
| --- | --- | --- |
| control_1 | 15724 | 3270 |
| control _2 | 16023 | 3280 |
| control _3 | 15651 | 3260 |
| control _4 | 15746 | 3264 |
| HA_1 | 15901 | 3276 |
| HA _2 | 16027 | 3310 |
| HA _3 | 16010 | 3303 |
| HA _4 | 16121 | 3342 |
| HC _1 | 16153 | 3355 |
| HC _2 | 16104 | 3363 |
| HC_3 | 16077 | 3318 |
| HC_4 | 16031 | 3325 |
| HCHA1_1 | 15911 | 3267 |
| HCHA1_2 | 15857 | 3274 |
| HCHA1_3 | 15851 | 3234 |
| HCHA1_4 | 15711 | 3284 |
| HCHA2_1 | 16250 | 3312 |
| HCHA2_2 | 16176 | 3289 |
| HCHA2_3 | 16110 | 3285 |
| HCHA2_4 | 16045 | 3238 |

**Table S2** GeneBank ID of study organisms.

| species | name | Genebank ID |
| --- | --- | --- |
| bacteria | *Vibrio astriarenae* | GCF_010587385.1 |
| bacteria | *Vibrio antiquarius* | GCF_000024825.1 |
| bacteria | *Vibrio mimicus* | GCF_000176375.1 |
| algae | *Porphyridium purpureum* | [GCA_000397085.1](https://ftp.ncbi.nlm.nih.gov/genomes/genbank/plant/Porphyridium_purpureum/latest_assembly_versions/GCA_000397085.1_Porphyridium_purpureum/) |
| algae | *Symbiodinium pilosum* | GCA_905231905.1 |
| sponge | *Amphimedon queenslandica* | GCF_000090795.2_v1.1 |
| sponge | *Ephydatia muelleri* | GCA_013339895.1 |
| cnidarian | *Acropora digitifera* | GCA_014634065.1 |
| cnidarian | *Hydra vulgaris* | GCF_022113875.1 |
| cnidarian | *Desmophyllum pertusum* | [GCA_029204205.1](https://www.ncbi.nlm.nih.gov/assembly/16210761) |
| cnidarian | *Pocillopora damicornis* | GCA_003704095.1 |
| cnidarian | *Porites lobata* | GCA_942486035.1 |
| snails | *Biomphalaria glabrata* | GCF_947242115.1 |
| snails | *Pomacea canaliculata* | GCF_003073045.1 |
| snails | *Gigantopelta aegis* | GCF_016097555.1 |
| fish | *Amphiprion percula* | [GCA_003047355.2](https://www.ncbi.nlm.nih.gov/assembly/2124461) |
| fish | *Danio rerio* | GCF_000002035.6 |
